# Upregulation of *miR-200c* and *miR-429* Suggests Reversal Towards Epithelial State in Venous Tumour Thrombus of Clear Cell Renal Cell Carcinoma

**DOI:** 10.3390/ijms26167951

**Published:** 2025-08-18

**Authors:** Tanja Čugura, Emanuela Boštjančič, Jera Jeruc

**Affiliations:** Institute of Pathology, Faculty of Medicine, University of Ljubljana, 1000 Ljubljana, Slovenia; tanja.cugura8@gmail.com (T.Č.); emanuela.bostjancic@mf.uni-lj.si (E.B.)

**Keywords:** renal cell carcinoma, venous tumour thrombus, epithelial–mesenchymal transition, *miR-200* family, cadherins, *TWIST1*, *SNAI2*

## Abstract

Renal cell carcinoma (RCC) has a well-established propensity to form grossly visible tumour thrombi; however a comprehensive understanding of the underlying mechanisms is still lacking. The epithelial–mesenchymal transition (EMT) has been implicated in the progression of many carcinomas, including RCC; however, its exact role in the formation of venous tumour thrombi remains unclear. This study aims to explore the involvement of the EMT in venous invasion in RCC. In 14 patients with WHO/ISUP grade 2/3 clear cell RCC with venous invasion, the expression of main EMT markers (the *miR-200* family, *miR-205*, *SNAI1*/*2*, *TWIST1*, *ZEB2*, and *CDH1*) was analyzed by qPCR in the selected tumour regions—the tumour centre (TC), the tumour periphery (TP), the venous tumour thrombus (VTT)—and compared to the corresponding non-neoplastic kidney tissue (N). Expression of E-cadherin, N-cadherin, and *ZEB2* was analyzed immunohistochemically. The *miR-200* family was downregulated in all areas examined compared to the corresponding N. When comparing the VTT with the TC, upregulation of *miR-200c* and *miR-429* was observed. *CDH1* was downregulated when the TP was compared with N, while *SNAI2* was downregulated in all tumour regions. There was a strong correlation between the expression of all members of the *miR-200* family. Our results demonstrate the presence of distinct molecular signatures between the selected ccRCC regions. The upregulation of two miRNAs in the VTT compared to the TC and their correlation with *CDH1* expression could indicate a reversal of the EMT towards a more epithelial cell state in the VTT.

## 1. Introduction

Clear cell renal cell carcinoma (ccRCC) is the most common subtype of renal cell carcinoma (RCC) and usually manifests in the 6th–7th decade of life [1,2]. The main prognostic factors are the WHO/ISUP (World Health Organization/International Society of Urological Pathology) nuclear grade and the TNM stage [3]. The WHO/ISUP grade is based on nucleolar prominence and cell pleomorphism, while the tumour stage is determined by the tumour extent (T), lymph node spread (N), and presence of metastasis (M). Most patients are diagnosed with organ-confined disease; however up to 30% of newly diagnosed patients already have metastatic disease. In addition, 30% of initially non-metastatic RCCs eventually develop distant metastases [4]. The prognosis of metastatic RCC is dismal, with a 5-year overall survival of 15% [5]. Despite the immense progress in the field of immunotherapy [6] and targeted therapies [7], metastatic ccRCC remains notoriously resistant to treatment options [8,9]. ccRCC most commonly metastasises to the lung, bone, and liver while also having a propensity to metastasise to unusual anatomical locations [10,11,12]. Unlike other types of carcinomas [13], lymphatic invasion is not the primary route of metastasis in ccRCC. Instead, ccRCC typically invades through veins, often with grossly evident tumour thrombi (TTs) extending into the renal vein and its branches at the renal sinus [14]. Interestingly, not all ccRCC patients with vascular invasion will develop metastases [15]. In order to disseminate, tumour cells must first gain access to the lymphatic or blood vasculature, survive and migrate through lymphovascular channels, and eventually extravasate at distant locations to form metastatic masses.

In carcinomas, the induction of the epithelial–mesenchymal transition (EMT) is strongly associated with tumour invasion. Through the EMT, cells lose their epithelial (E) characteristics and gain mesenchymal (M) traits, becoming spindle, motile cells that express mesenchymal cadherins (N-cadherins) [16]. The EMT is a dynamic, highly plastic process, which generates cells co-expressing E and M markers. These cells undergo the so-called partial EMT (pEMT) and exhibit a mixed, hybrid, E/M phenotype with enhanced invasive potential [17,18]. Indeed, increasing evidence shows that the acquisition of a limited set of mesenchymal features with retention of certain epithelial features (i.e., pEMT) generates cancer cells with greater invasive and metastatic properties than the full EMT [19]. The reverse process, the mesenchymal–epithelial transition (MET), seems to enable metastatic colonization through the re-differentiation of tumour cells, allowing the formation of secondary tumour masses [20,21]. A variety of EMT transcription factors (EMT-TFs) [22], microRNAs (miRNAs) [23], and epigenetic mechanisms [24] can regulate the EMT/MET. Our previous study supports the notion that a partial EMT, particularly *miR-200* downregulation, is implicated in the progression of ccRCC to sarcomatoid RCC [25].

We therefore hypothesized that a partial EMT might also be involved in vascular invasion of ccRCC. In the present study, we analyzed several EMT markers to elucidate the possible role and degree of EMT activation in renal vein invasion of ccRCC.

## 2. Results

### 2.1. Patients, Tissue Samples, and Follow-Up

In total we analyzed 59 tissue samples from 14 patients with ccRCC with invasion into the renal vein. The gender and age of patients as well as the TNM stage and tumour characteristics are listed in Table 1.

Follow-up data were available for 12 patients. Four patients were primarily diagnosed with metastatic RCC. One of them is still alive at the time of writing the manuscript, while the other three died 1, 3 and 4 years after their diagnosis, respectively. Of the eight patients who initially presented with localized disease, all eventually developed metastatic disease, with a median time to metastasis of 1.4 years. Five patients are still alive at the time of writing, while the remaining three patients died on average 4 years after their RCC diagnosis. As all cases were metastatic, no correlation analysis was performed with the expression of miRNAs and mRNAs.

### 2.2. Morphology and Immunohistochemical Expression of EMT-Related Markers

E-cadherin staining was preserved in all samples of non-neoplastic renal tissue. Membranous, circumferential E-cadherin staining was present in Bowman’s capsule, distal tubules, and collecting ducts, while N-cadherin staining was expressed in proximal tubules and the thin limb of the loop of Henle.

**Figure 1:**
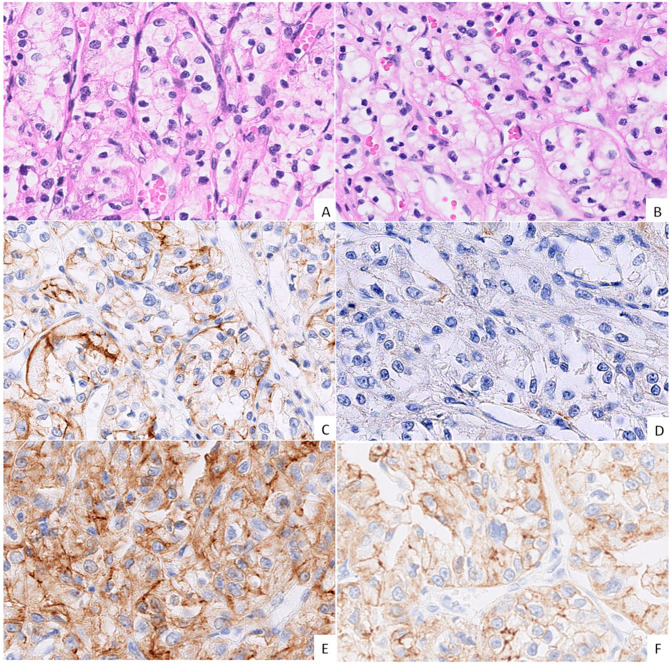
Morphology and immunohistochemical expression (IHC) of cadherins in the tumour centre (TC) and the tumour periphery (TP). ((**A**,**B**); H&E, 40× magnification, grade 2 ccRCC) Morphologically indistinguishable TC (**A**) and TP (**B**) regions of the same tumour; classical ccRCC morphology of epithelioid cells with clear cytoplasm forming nests and cords with a well-vascularized stroma. ((**C**,**E**); IHC, 40× magnification, grade 3 ccRCC) The TC showing moderate, uneven membranous E-cadherin expression (**C**), with similar co-expression of N-cadherin (**E**). ((**D**,**F**); IHC, 40× magnification, grade 3 ccRCC) The TP shows negative E-cadherin expression (**D**), while N-cadherin expression is of moderate intensity (**F**).

**Figure 2:**
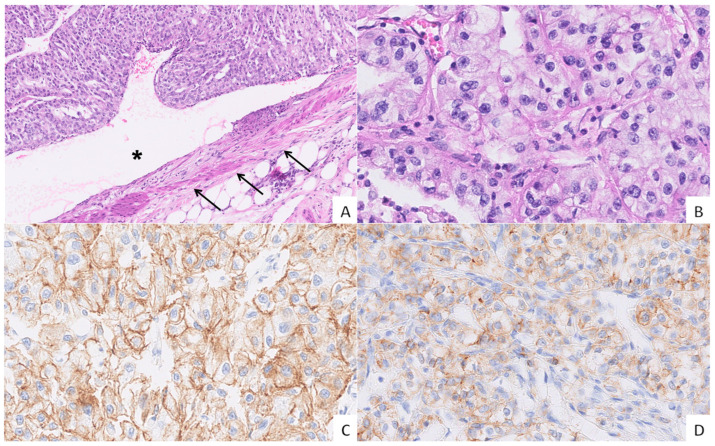
Vascular invasion in grade 3 ccRCC and the expression of cadherins. ((**A**), H&E, 10× magnification) A venous tumour thrombus (VTT) occluding the lumen (asterisk) of a renal vein (arrows). ((**B**), H&E, 40× magnification) The VTT showing an epithelioid morphology with abundant optically clear cytoplasm, round nuclei, and punctate nucleoli. ((**C**), IHC, 40× magnification) Moderate to strong membranous E-cadherin expression in tumour cells. ((**D**), IHC, 40× magnification) Moderate membranous N-cadherin expression in the majority of tumour cells.

In the tumour centre (TC), membranous expression of N-cadherin was strong in 71% of the cases, whereas the expression of E-cadherin was reduced in 64% of the cases. ZEB2 staining was absent in all TC samples. In the tumour periphery (TP), E-cadherin expression was decreased in 79% of the samples and ranged from moderate to weak to absent, while strong N-cadherin expression was maintained in half of the cases. ZEB2 was focally expressed in a minority of TP samples (14%). In a venous tumour thrombus (VTT), E-cadherin staining was reduced in 64% of the cases and ranged from moderate to weak, while strong expression was maintained in five samples. The decrease in N-cadherin expression was most pronounced in the VTT compared to the TC and TP, as strong expression was present in only 42% of the cases. ZEB2 expression was absent in all VTT samples. The results are shown in Table 2 and Figure 1 and Figure 2.

### 2.3. Quality Control of Isolated RNA and the Expression of Reference Genes

All samples passed the initial quality control and showed successful amplification of 100 bp long fragments of the house-keeping gene *GAPDH* (Cq < 35). We used suggested reference genes (RGs) for miRNAs (*miR-28*, *miR-103a-3p*, and *miR-106a-5p*) and mRNAs (*ACTB*, *B2M*, *HPRT1*, *RPL13*, and *SDHA*) as previously described [25]. All successfully amplified RGs for both miRNAs (three reference miRNAs) and mRNAs (five RGs) were stably expressed in 12 out of 14 ccRCCs and the corresponding non-neoplastic kidneys. Two cases showed high variability in the RGs of mRNAs/miRNAs and were omitted from further analyses.

### 2.4. Expression of miRNAs

#### 2.4.1. Expression of the *miR-200* Family and *miR-205* in the Tumour Centre, Tumour Periphery, and Renal Vein Tumour Thrombus Compared to a Non-Neoplastic Kidney

**Figure 3:**
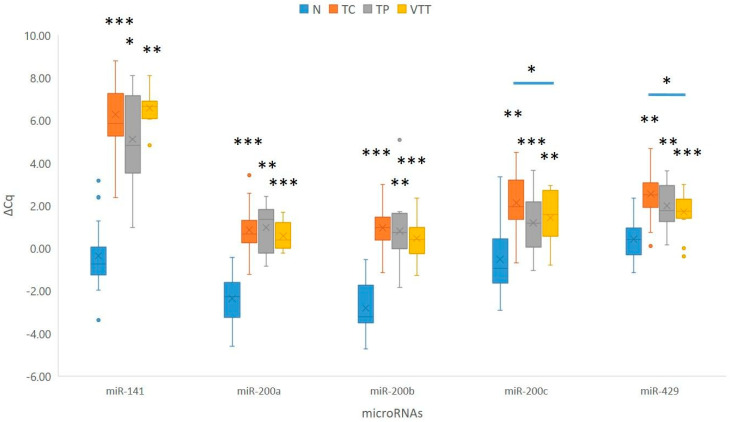
Expression of the *miR-200* family in the TC, TP, and VTT in comparison to the corresponding normal renal cortex (N). *, *p* < 0.05; **, *p* < 0.01; ***, *p* < 0.001; Cq, quantitation cycle; ΔCq, delta Cq; N, normal renal cortex; TC, tumour centre; TP, tumour periphery; VTT, venous tumour thrombus.

When compared to adjacent non-neoplastic renal cortical tissue, statistical analysis showed statistically significant downregulation of the *miR-200* family in the TC (*p* < 0.001 for *miR-141* and *miR-200a*/*b*; *p* = 0.001 for *miR-200c* and *miR-429*), in the TP (*p* = 0.034 for *miR-141*; *p* = 0.003 for *miR-200a* and *miR-429*; *p* = 0.001 for *miR-200b*; *p* = 0.006 for *miR-200c*), and in the VTT (*p* = 0.002 for *miR-141* and *miR-200c*; *p* = 0.001 for *miR-200a*/*b* and *miR-429*). *miR-205* did not show a statistically significant change in expression. Results are summarized in Figure 3 and Table S1.

#### 2.4.2. Expression of the *miR-200* Family and *miR-205* in the Renal Vein Tumour Thrombus Compared to the Tumour Centre and Tumour Periphery

When compared to the TP, none of the analyzed miRNAs showed statistically significant deregulation of miRNAs in the VTT. However, *miR-200a* showed borderline upregulation in the VTT when compared to the TP (*p* = 0.06). When the VTT was compared to the TC, two of the analyzed miRNAs showed statistically significant upregulation in the VTT (*p* = 0.031 for *miR-200c* and *p* = 0.020 for *miR-429*). Furthermore, we observed statistically borderline up-regulation of *miR-141* (*p* = 0.05) at the TP when compared to the TC. Results are summarized in Figure 3.

### 2.5. Expression of EMT-TFs and E-Cadherin in the Tumour Centre, Tumour Periphery, and Renal Vein Tumour Thrombus Compared to a Corresponding Non-Neoplastic Kidney

**Figure 4:**
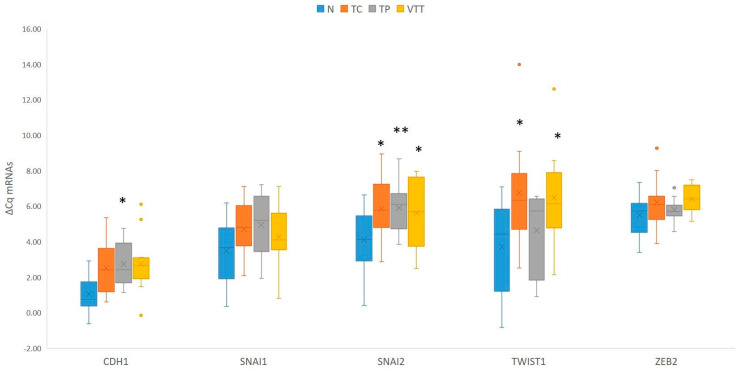
Expression of EMT-TFs and *CDH1* in the TC, TP and VTT in comparison to the corresponding normal renal cortex (N). *, *p* < 0.05; **, *p* < 0.01; *CDH1*, the gene encoding E-cadherin; Cq, quantitation cycle; ΔCq, delta Cq; N, normal renal cortex; TC, tumour centre; TP, tumour periphery; VTT, venous tumour thrombus.

When compared to adjacent non-neoplastic renal cortical tissue, statistical analysis showed significant downregulation of *CDH1* in the TP (*p* = 0.028) and borderline downregulation in the TC and VTT (*p* = 0.053 and *p* = 0.066, respectively). While *SNAI1* showed a borderline significant change in expression, *SNAI2* showed statistically significant downregulation in all locations, namely the TC, TP and VTT (*p* = 0.015, *p* = 0.008, and *p* = 0.021, respectively). Moreover, *TWIST1* was borderline downregulated in the TC (*p* = 0.05) and significantly downregulated in the VTT (*p* = 0.025), with no significant change in expression in the TP. Results are summarized in Figure 4 and Table S1. When EMT-TFs and E-cadherin in the VTT were compared either to the tumour centre or tumour periphery, no significant change in expression was observed in any analyzed mRNA.

### 2.6. Correlation Between the miR-200 Family, Its Targets E-Cadherin, and EMT-TFs

**Figure 5:**
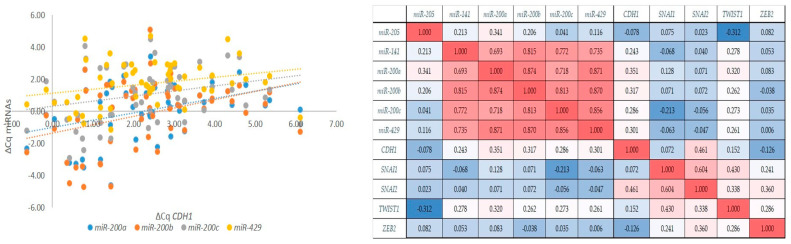
Correlations between markers of the EMT. Correlations between the expression of the *miR-200* family and the expression of *CDH1* (**left**); a graphical correlation matrix between EMT markers (**right**). Legend: *CDH1*, the gene encoding E-cadherin; Cq, quantitation cycle; ΔCq, delta Cq; red colour, positive correlation; blue colour, negative correlation (intensity of red and blue increases with number increasing or decreasing towards 1 or −1, respectively).

As expected, we observed a strong to very strong correlation between expressions of all members of the *miR-200* family (r_s_ > 0.69, *p* < 0.001). However, *miR-200a*/*b*/*c* and *miR-429* also showed a weak correlation with the expression of *CDH1* (r_s_ > 0.29, *p* < 0.05). Moreover, *CDH1* showed a moderate correlation with *SNAI2* (r_s_ = 0.46, *p* < 0.001), and *SNAI1* showed a moderate correlation with *SNAI2* and *TWIST1* (r_s_ = 0.60, *p* < 0.001; r_s_ = 0.43, *p* = 0.004, respectively); *SNAI2* showed a weak correlation with *TWIST1* and *ZEB2* (r_s_ = 0.33, *p* = 0.033; r_s_ = 0.360, *p* = 0.013). Results are summarized in Figure 5.

## 3. Discussion

Although renal vein invasion is a recognized adverse prognostic factor for the survival of RCC patients, there is relatively little research on this topic. A recent bibliometric analysis of the literature on RCC with VTTs has shown an upward trend in this area of research over the past decade [26]. However, research has mainly focused on prognostic significance, surgical management of venous tumour thrombi, and, more recently, immune checkpoint inhibitor therapies. The molecular mechanisms underlying venous invasion are therefore still poorly understood. In the present study, we analyzed the expression of various EMT-associated markers to investigate their potential role during venous invasion of ccRCC. When investigating the expression of the *miR-200* family, we first observed a downregulation of all members in all investigated areas, namely in the centre of the tumour, the periphery of the tumour, and in the renal vein tumour thrombus, when compared to the corresponding renal cortical tissue. Surprisingly, when comparing the expression of *miR-200* in the VTT with the expression in the tumour itself, an upregulation of *miR-200c* and *miR-429* was observed in the VTT compared to the TC. In addition, *SNAI2* was downregulated in all examined areas. Second, we observed downregulation of *CDH1* in the TP and downregulation of *TWIST1* in the VTT and TC.

We observed no morphological differences in tumour cells between the TC, TP, and VTT. In all regions, the tumour cells consistently exhibited an epithelial morphology with no morphological evidence of a shift towards a spindled morphology. This finding aligns with previous reports on vascular invasion [27,28,29]. The nuclear grade in the TP and TC matched the tumour grade in the corresponding VTT in all cases. Interestingly, recent studies found discrepancies between the nuclear grade of the main tumour mass and the VTT in ccRCC [14,30]. Indeed, the VTT was not always composed of cells with the highest nuclear grade, suggesting that a higher nuclear grade may not correlate with the degree of invasiveness. In addition, the nuclear grade in the VTT and not the overall tumour nuclear grade seems to be the most robust predictor of time to metastasis [14].

When evaluating E-cadherin and N-cadherin expression immunohistochemically, there was a notable decrease in E-cadherin expression in the TC, compared to its expression in the proximal tubules of a normal kidney, while N-cadherin expression remained mostly strong. The VTT showed almost identical E-cadherin expression profiles as the TC, while N-cadherin expression was decreased compared to the TC. The decrease in E-cadherin expression was most pronounced in the TP, while N-cadherin expression in the TP was similar to that in the VTT. ZEB2 was focally expressed in 14% of TP samples, while being negative in the remainder of TPs and all TC and VTT samples. These diverse cadherin signatures between the analyzed regions implicate the possibility of the occurrence of distinct hybrid states on the EMT-MET spectrum in the same tumour, ranging from a more mesenchymal phenotype (i.e., TP) to an epithelial phenotype (i.e., VTT). Hybrid or partial EMT states may enable tumour cells to acquire motility while retaining the benefits of tumour cell cohesion, enabling invasion in the form of cell clusters in a process called collective invasion. It has been shown that collective metastasis enables tumour cells to colonize secondary sites more efficiently, resist cell death, and evade the immune system compared to single-cell invasion [31]. Indeed, a number of studies have shown that the so-called leading cells in collective cell migration occupy a hybrid-EMT state as they express some, but not all, of the hallmark EMT transcription factors in combination with typical epithelial markers. However, the composition of EMT transcriptional programmes expressed by leading cells varies widely between cancer types and subtypes [32].

There was a strong correlation between the expression of all members of the *miR-200* family, suggesting their cooperative action during the EMT and MET [33]. The expression of *miR-200c* and *miR-429* was upregulated in the VTT compared to the TC. The *miR-200* family has a broad physiological role; however, it mainly serves to maintain the overall epithelial phenotype in cells by inhibiting *ZEB1* and *ZEB2*. In several carcinomas, the *miR-200* family is downregulated, allowing the activation of the EMT process, which enables tumour cells to obtain invasive and metastatic properties [34]. During venous invasion, tumour cells breach the vascular wall, enter the blood circulation, and form tumour clusters [35]. ccRCC has an established propensity to invade renal veins in the form of large and oftentimes macroscopically visible VTTs [8], consistent with collective metastasis [31]. The upregulation of two of the analyzed members of the *miR-200* family in the VTT compared to the TC could indicate the transition of VTT cells towards the MET. Indeed, the tumour cells in the VTT maintain a strikingly epithelioid morphology, with no evidence of spindling, which corroborates other studies on collective cell migration in tumours (reviewed in [31,36]). The MET is believed to participate in the establishment and stabilization of distant metastases by allowing cancerous cells to regain epithelial properties at metastatic sites. However, it has already been suggested in a previous study that the MET is an early event in the metastatic process [37]. A recent study using integrative transcriptome and proteome analyses of RCC with a VTT found *KLF4* to be one of the differentially expressed genes in the VTT compared to a primary tumour [38]. *KLF4* belongs to a group of recently described MET transcription factors (MET-TFs) that promote epithelization by direct transcriptional repression of mesenchymal markers and by establishing mutual bidirectional inhibitory circuits with EMT-TFs [39].

Among *miR-200* family members, *miR-200b*/*c* act as key switches between EMT differentiation and proliferation. *miR-429*, on the other hand, in addition to its role in the EMT, appears to be critical for the regulation of cellular adaptation to hypoxia. A recent study by Zheng et al. [40] showed that in endometriosis, an estrogen-dependent disorder similar to malignant tumours regarding biological behaviour, *miR-429* facilitates proliferation, migration, and invasiveness of endometrial stromal cells by modulating the HIF1A inhibitor-mediated HIF1A/VEGF signalling pathway. The molecular alterations in the VHL/HIF pathway are well documented in ccRCC. Loss of the tumour suppressive role of the VHL protein (pVHL) leads to accumulation and constitutive activation of hypoxia inducible factor 1α (HIF-1α), which is responsible for the metabolic switch that allows survival of cells in the hypoxic environment [41,42]. Increased levels of HIF-1α are usually associated with a worse prognosis [43]. It is possible that in venous invasion of RCC, *miR-429* not only has a role in EMT regulation but is also a crucial modulator of HIF signalling. Indeed, several studies have shown that *miR-429* regulates HIF-1α expression in endothelial cells [44,45,46]. Additionally, Xu et al. demonstrated that *miR-429* negatively regulates the VEGF pathway in hypoxia-induced retinal neovascularization, highlighting its significance in this process [47]. Ge et al. further provided evidence that *HIF-1α* is a target gene of *miR-429* in amniotic cells [45]. Additionally, Ye et al. identified *miR-429* as a regulator of the VEGFA pathway in ovarian cancer tissue, highlighting its potential as a future diagnostic and therapeutic biomarker [48]. Furthermore, Zhu et al. confirmed that *miR-429* targets the 3′-UTR of proline-hydroxylase-2 (*PHD2*) mRNA, demonstrating that inhibition of *miR-429* significantly increased *PHD2* mRNA levels while decreasing HIF-1α levels [49]. Taken together, these results provide evidence of a negative feedback loop between *miR-429* and the HIF1A pathway as well as its involvement in the VEGF pathway.

Among EMT-TFs *SNAI2* showed significant downregulation in all the analyzed compartments, namely the TC, TP, and VTT compared to N. This is in contrast to our previous study where no significant differences in EMT-TF expression were found between normal renal tissue and low-stage RCC [25]. However, our results are in line with the study by Mikami et al., showing that SNAI2 protein expression levels negatively correlate with the pathological tumour stage, suggesting that *SNAI2* was downregulated in advanced RCCs [50]. Additionally, *TWIST1* was significantly downregulated in the VTT and showed borderline downregulation in the TC compared to N. *TWIST1*, a basic helix-loop-helix transcriptional factor expressed in various types of carcinomas, is a key player in tumour metastasis by inducing the EMT [51]. Elevated *TWIST1* expression in primary tumours is frequently linked to a poor prognosis and increased metastatic potential. Studies have found that higher levels of *TWIST1* correlate with more aggressive diseases, earlier relapse, and a greater likelihood of distant metastasis [52,53,54]. A report by Tsai et al. also indicated that turning off *TWIST1* reversed the EMT process, leading to the subsequent occurrence of the MET, enabling colonization and the formation of metastases [55]. Furthermore, a study by Yang et al. on human hypopharingeal and lung carcinoma cell lines with *HIF-1α* overexpression showed that small interfering RNAs (siRNAs) can repress *TWIST1*, causing a shift towards the MET with the switch from mesenchymal (N) to epithelial (E) cadherin expression [56]. The downregulation of *TWIST1* in the VTT along with preserved E-cadherin expression and a reduction in N-cadherin expression could indicate a transition towards the MET during venous invasion. Taken together, these results indicate an important role of *TWIST1* as a regulator of epithelial plasticity during cancer metastasis.

There are several advantages and limitations of our study. The main advantage is the use of the punching technique, which allowed us to obtain tumour samples at the locations of interest, as determined by microscopic analysis. Comparing different locations within the same tumour (i.e., TC and TP) and matching VTT from the same patient is important for evaluating the expression of miRNAs, as their expression can be tissue-specific and is also influenced by gender and age [57]. The limitation of our study is the small number of patients, thus limiting the statistical power. However, only samples that successfully passed the initial quality control and samples with stable expression of the RGs were selected for further analysis, which limited the number of included samples. Nevertheless, tumours were quite homogeneous in terms of primary tumour stages and grades. As all cases were metastatic, the expression of EMT markers in correlation with clinical outcomes was therefore not possible. To the best of our knowledge, this is the first study comparing the expression of different EMT markers in distinct regions of ccRCC with a venous tumour thrombus.

In summary, this study demonstrates heterogeneity in the expression of EMT markers between different tumour regions, indicating subtle differences in cell states in the EMT-MET spectrum. These hybrid intermediate states could confer phenotypic plasticity to cells, enabling invasion and tumour progression. As the EMT is a highly complex process, our results may contribute to the understanding of the intricate mechanisms associated with tumour thrombus formation and metastasis initiation in ccRCC. Further studies focusing on the analysis of single cells and larger EMT marker panels are needed to further explore the process of venous invasion in ccRCC. miRNAs have emerged as promising therapeutic targets, as modulating their expression may offer a powerful approach in limiting disease progression [58]. In addition, the prevention or reversing the effects of EMT is increasingly regarded as a promising strategy in the development of novel cancer therapies [59]. However, this remains challenging due to potential serious and unpredictable side effects. While anti-EMT interventions may suppress the migratory and invasive properties of primary tumours, they may also promote the MET, thereby facilitating metastatic colonization. Therefore, studying the expression patterns of EMT-TFs and their regulatory mRNAs across different tumour types and stages may help in guiding more effective and context-specific therapeutic strategies.

## 4. Materials and Methods

### 4.1. Patients and Tissue Samples

**Figure 6:**
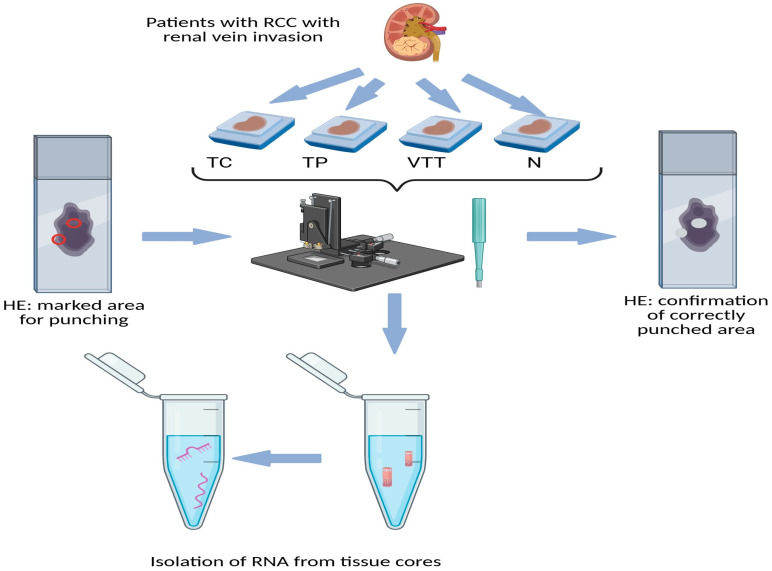
Schematic representation of obtaining tissue cores (punching technique) from FFPE tissue of RCC. Legend: HE, haematoxylin and eosin; N, morphologically normal renal tissue; RCC, renal cell carcinoma; TC, tumour centre; TP, tumour periphery; VTT, venous tumour thrombus; red circle on the left slide, marked punching area; white spots on right slide, confirmation of correctly punched area.

This retrospective analysis included 14 patients diagnosed with renal cell carcinoma exhibiting invasion of the renal vein, all of whom underwent surgical treatment between 2017 and 2022. None of the patients had received chemotherapy, radiotherapy, or renal artery embolization prior to their surgical intervention. TNM staging was conducted according to the most recent TNM classification for renal tumours [60]. Following nephrectomy, the resection specimens were processed in accordance with standard protocols. The specimens were fixed in 10% buffered formalin for 24 h, after which representative samples were taken from both the tumour and the macroscopically normal renal cortex. The tissue samples were then embedded in paraffin (formalin-fixed paraffin-embedded, FFPE), sliced into sections 3–4 µm thick, and stained with haematoxylin and eosin. All slides were re-evaluated by a pathologist specializing in urologic pathology (J.J). Representative slides were chosen from the tumour centre (TC), tumour periphery (TP), renal vein tumour thrombus (VTT), and adjacent non-neoplastic renal cortical tissue (N). Corresponding paraffin blocks for all slides were retrieved from the archives of the Institute of Pathology, Faculty of Medicine, University of Ljubljana. Using a microneedle puncture technique, the tissue for molecular studies was acquired from the paraffin blocks utilizing a 0.6 mm needle, obtaining 3–5 tissue cores from each TC, TP, VTT, and N. Figure 6 represents the punching technique. A more detailed representation is available in one of our previous studies [61].

### 4.2. Immunohistochemistry

FFPE tissue blocks were sectioned into 4 µm thick slices for immunohistochemical staining. All reagents were sourced from Ventana (Tucson, AZ, USA). Commercially available antibodies for E-cadherin (Dako, Santa Clara, CA, USA, M3612, clone KS 20.8, dilution: 1:20), N-cadherin (Abcam, Cambridge, UK, AB225719, dilution: 1:30), and ZEB2 (Abcam, Cambridge UK, AB223688, dilution: 1:250) were utilized. Deparaffinization, antigen retrieval, and staining were conducted using the BenchMark XT automatic immunostainer (Ventana, Tucson, AZ, USA) with ultraVIEW for E-cadherin and the OptiVIEW detection system for N-cadherin and ZEB2. Appropriate positive and negative internal tissue controls were implemented to ensure accurate immunohistochemical evaluation. A semi-quantitative method was applied to assess the extent of staining (negative, 0; <30%, +; 30–60%, ++; >60%, +++) and the staining pattern (nuclear, cytoplasmic, or membranous). IHC staining results were assessed by two pathologists (T.Č. and J.J.). To minimize inter-observer variability, appropriate negative and positive reaction controls were employed, and strict adherence to the established scoring system and cut-off values was maintained. In challenging cases, which constituted a small fraction of the analyzed samples, a consensus was reached through mutual viewing and scoring of the slides.

### 4.3. RNA Isolation from FFPE Tissue Samples

For RNA isolation, 3–5 microneedle punches (0.6 mm) were performed at each site on the FFPE blocks. To confirm the accuracy of the obtained tissue cores, additional slides were prepared from the punched FFPE blocks, stained with HE, and evaluated (Figure 6). Total RNA was manually isolated using the MagMAX FFPE DNA/RNA Ultra kit (Thermo Fisher Scientific, Austin, TX, USA), following the manufacturer’s instructions, with one modification for protease digestion, which was performed overnight. The reagents used were from Thermo Fisher Scientific (Austin, TX, USA), except for ethanol (Merck KGaA, Darmstadt, Germany) and the deparaffinization solution (Xylene; Sigma-Aldrich; Merck KGaA, Darmstadt, Germany). The concentration and purity of the RNA isolates were measured using a NanoDrop-One machine (Thermo Fisher Scientific; Foster City, CA, USA) at wavelengths of 230, 260, and 280 nm.

For quality control, reverse transcription (RT) was conducted as outlined in Section 4.5.1, followed by the amplification of *GAPDH* (Hs_GAPDH_vb.1_SG, 100 bp, Qiagen, Venlo, The Netherlands) utilizing SybrGreen technology through quantitative real-time PCR (qPCR). All samples included in the study underwent this initial quality control assessment. Samples that did not amplify (Cq < 35) were excluded from further analysis.

### 4.4. Analysis of Expression of the miR-200 Family

#### 4.4.1. Reverse Transcription (RT) of miRNAs

Isolated RNA was transcribed into cDNA using the miRCURY LNA RT Kit (Qiagen; Hilden, Germany) according to the manufacturer’s instructions. The successful reverse transcription (RT) was validated by the addition of spike-in RNA (UniSp6) followed by its quantification. The 10 µL reaction master mix consisted of 2 µL of the 5× miRCURY RT Reaction Buffer, 1 µL of the 10× miRCURY RT Enzyme Mix, 0.5 µL of UniSp6 Spike-in, and 6.5 µL of total RNA (10 ng). The reaction was carried out under the following conditions: incubation at 42 °C for 60 min, followed by heat inactivation at 95 °C for 5 min and immediate cooling to 4 °C.

#### 4.4.2. Quantitative Real-Time PCR (qPCR)

qPCR utilizing SYBR Green technology was conducted to evaluate the expression levels of the *miR-200* family. Amplification efficiency was obtained from a prior publication [25]. For quantification, qPCR was performed using a pre-designed mixture of primers specific to miRNA expression. The miRCURY LNA SYBR GREEN Kit (Qiagen; Venlo, The Netherlands) was employed to analyze the selected miRNAs, namely *miR-141*, *miR-200a*, *miR-200b*, *miR-200c*, *miR-205*, and *miR-429*, relative to the geometric mean of the reference miRNAs suggested by other authors (*miR-28*, *miR-103a-3p*, and *miR-106a-5p*) [54]. In brief, 3 µL of diluted (1:60) cDNA was added to a 10 µL reaction volume containing 1 µL of 10× miRCURY LNA miRNA PCR Primer Assay, 5 µL of SybrGreen MasterMix, and 1 µL of PCR-grade H_2_O. The thermal cycling conditions included an initial denaturation process at 95 °C for 2 min, followed by 40 cycles of denaturation at 95 °C for 10 s and annealing and amplification at 56 °C for 1 min, concluding with a melting curve analysis on a Rotor Gene Q. All qPCR reactions were conducted in duplicates.

### 4.5. Analysis of the Expression of EMT-Related Genes

#### 4.5.1. Reverse Transcription (RT) for mRNAs

For the reverse transcription of isolated mRNA, the OneTaq ^®^ RT-PCR Kit (New England Biolabs, Ipswich, MA, USA) was utilized, employing a combination of random hexamers and oligo-dT primers as per the manufacturer’s protocol. The maximum quantity of RNA was used in a total 10 µL RT reaction, which included 1 µL of random hexamers incubated for 5 min at 70 °C. Subsequently, 5 µL of the reaction mix and 1 µL of the enzyme mix were added, followed by incubation at 25 °C for 5 min, 42 °C for 1 h, and a final step at 80 °C for 5 min.

#### 4.5.2. Quantitative Real-Time PCR (qPCR) and Probes

The TaqMan-based approach (Thermo Fisher Scientific, Waltham, MA, USA) was employed for the qPCR methodology. A pre-designed mixture of primers and probes was utilized for the expression analysis of mRNAs relative to the selected RGs, chosen based on their suitability for normalizing mRNA levels in FFPE tissue samples from patients with RCC [62]. Five RGs were selected, *ACTB* (Hs01060665_g1), *HPRT1* (Hs02800695_m1), *RPL13A* (Hs03043885_g1), *SDHA* (Hs00188166_m1), and *B2M* (Hs 99999907_m1), as previously described [25]. Prior to qPCR amplification, efficiencies were obtained from earlier studies [25].

The expression analyses involved 10 µL reactions that included 5.0 µL of FastStart ™ PCR Master mix (Roche Diagnostics, Basel, Switzerland), 0.5 µL of the TaqMan probe, and 4.5 µL of cDNA (1.5 ng/reaction). The cycling protocol consisted of 50 °C for 2 min, 95 °C for 10 min, followed by 40 cycles of 95 °C for 15 s and 62 °C for 1 min. We analyzed the expression of the E-cadherin gene (*CDH1*) (Hs01023895_m1), *SNAI1* (Hs00195591_m1), *SNAI2* (Hs00161904_m1), *TWIST1* (Hs01675818_s1), and *ZEB2* (Hs01095318_m1) in relation to RGs that were successfully amplified and consistently expressed across all samples analyzed. The amplicon length for all analyzed mRNAs, the gene of interest, and RGs was maintained below 100 bp according to initial quality control with *GAPDH*. Notably, *TWIST2* (Hs00382379_m1) and *ZEB1* (Hs03680599_m1), two EMT transcription factors, were excluded due to their unsuccessful amplification in our previous study.

### 4.6. Statistical Analysis

Results were expressed as relative gene expression using the ΔCq method. All Cq values were corrected for PCR efficiencies, and the expression of the gene of interest (CqGOI) was calculated relative to a geometric mean of reference genes (CqRG) to yield ∆Cq. For comparing miRNA expression differences between paired samples (e.g., TC and TP), the ∆Cq values and the Wilcoxon rank test were employed. Spearman’s rank-order correlation was used for all correlations and associations. Statistical analysis (including power analysis) was conducted using SPSS version 27 (SPSS Inc., Chicago, IL, USA), with differences deemed statistically significant at *p*-values < 0.05.

## Figures and Tables

**Table 1:** Patients’ characteristics.

Male/female	13:1
Age (mean ± SD)	64.5 ± 7.2
Largest tumour diameter (mean in cm)	6.5
pTNM ^1^	pT3a N0 (*n* = 11) pT3a N1 (*n* = 2) pT3b N0 (*n* = 1)
WHO/ISUP grade	Grade 2 (*n* = 1) Grade 3 (*n* = 13)

^1^ Pathological TNM staging according to the 8th AJCC edition [3].

**Table 2:** Immunohistochemical expression of E-cadherin, N-cadherin, and ZEB2 in the selected regions expressed as the number of cases showing strong (+++), moderate (++), weak (+), or no (−) reaction. N, non-neoplastic kidney; TC, tumour centre; TP, tumour periphery; VTT, venous tumour thrombus.

	E-Cadherin				N-Cadherin				ZEB2			
	+++	++	+	−	+++	++	+	−	+++	++	+	−
N	14 * (100%)	0 (0%)	0 (0%)	0 (0%)	14 ** (100%)	0 (0%)	0 (0%)	0 (0%)	0 (0%)	0 (0%)	0 (0%)	14 (100%)
TC	5 (36%)	6 (43%)	1 (7%)	2 (14%)	10 (71%)	4 (29%)	0 (0%)	0 (0%)	0 (0%)	0 (0%)	0 (0%)	14 (100%)
TP	3 (21%)	8 (58%)	2 (14%)	1 (7%)	7 (50%)	4 (29%)	3 (21%)	0 (0%)	0 (0%)	0 (0%)	2 (14%)	12 (86%)
VTT	5 (36%)	6 (43%)	2 (14%)	1 (7%)	6 (43%)	4 (29%)	4 (29%)	0 (0%)	0 (0%)	0 (0%)	0 (0%)	14 (100%)

* In distal renal tubules. ** In proximal renal tubules.

## Data Availability

The raw data supporting the conclusions of this article will be made available by the authors upon request.

## Supplementary Materials

The following supporting information can be downloaded at: https://www.mdpi.com/article/10.3390/ijms26167951/s1.

## Abbreviations

The following abbreviations are used in this manuscript:

ccRCC	Clear cell renal cell carcinoma
CDH1	Gene encoding E-cadherin
EMT	Epithelial–mesenchymal transition
FFPE	Formalin-fixed paraffin-embedded
HIF1A	Hypoxia-inducible factor 1-alpha
MET	Mesenchymal–epithelial transition
miRNA	MicroRNA
N	Non-neoplastic kidney tissue
pEMT	partial EMT
RCC	Renal cell carcinoma
TC	Tumour centre
TF	Transcription factor
TP	Tumour periphery
VEGF	Vascular endothelial growth factor
VTT	Venous tumour thrombus
